# Medical vs. Organizational Complaints: A Machine Learning Analysis Reveals Divergent Patterns in Patient Reviews Across Russian Cities

**DOI:** 10.3390/healthcare13202641

**Published:** 2025-10-20

**Authors:** Irina Evgenievna Kalabikhina, Anton Vasilyevich Kolotusha, Vadim Sergeevich Moshkin

**Affiliations:** 1 Population Department, Faculty of Economics, Lomonosov Moscow State University, Moscow 119991, Russia; ikalabikhina@yandex.ru; 2 Department of Information Systems, Ulyanovsk State Technical University, Ulyanovsk 432027, Russia; v.moshkin@ulstu.ru

**Keywords:** machine learning, patient reviews, review classification, healthcare quality, logistic regression, natural language processing, patient satisfaction, Russia

## Abstract

**Background:** The growth of digital patient feedback presents a new opportunity for healthcare quality monitoring. This study addresses the need to automatically classify the content of patient reviews to identify primary sources of dissatisfaction. **Objective:** The purpose of this study is to develop a machine learning algorithm for classifying negative patient reviews into two core categories: medical content (M—pertaining to diagnosis, treatment, and outcomes) and organizational support (O—pertaining to logistics, cost, and communication). We aim to identify which type of concern prevails and to analyze variations across cities, patient gender, and medical specialties. **Methods:** A database of 18,680 negative patient reviews (rated 1 star) was compiled from the Russian aggregator infodoctor.ru for the period from July 2012 to August 2023. A training set was created using an independent annotation procedure with three experts. A logistic regression model was trained to classify reviews into M and O categories, demonstrating an accuracy of 88.5%. **Results:** The analysis revealed a significant structural shift in Moscow, where since 2021, medical (M) complaints began to prevail over organizational (O) ones. This trend was not observed in St. Petersburg or other major Russian cities. Notably, in St. Petersburg, M-type reviews were more common within the most represented medical specialties, whereas O-type reviews consistently dominated in other cities. Gender differences were most pronounced in St. Petersburg, where women were more frequently authors of M reviews and men of O reviews. **Conclusions:** The developed algorithm provides a valuable tool for the automated monitoring of patient feedback. It enables healthcare managers to distinguish between clinical and service-related issues, facilitating targeted improvements in medical service quality and patient satisfaction.

## 1. Introduction

The monitoring and analysis of patient reviews on social media and dedicated platforms have emerged as a valuable tool for healthcare management and quality assessment [1,2,3]. This approach enables the identification and categorization of patient complaints, which can be further enriched by considering the socio-demographic characteristics of both patients and doctors [4,5,6]. Such analysis is intrinsically linked to the development of digital demography and digital medicine, promising new research areas that leverage digital traces to understand population needs and behaviors [7,8].

The significance of this field is amplified in the context of global demographic shifts, particularly population aging. The growing share of older consumers of healthcare services and an aging physician workforce underscore the importance of understanding the specific needs and patterns within these groups [1,9]. A critical step in this process is the development of reliable, automated criteria for classifying patient complaints. A foundational distinction can be made between complaints related to medical content (M)—pertaining to diagnosis, treatment, and outcomes—and those concerning organizational support (O)—such as waiting times, cost, and staff communication [5,10,11]. This distinction is crucial because these two complaint types necessitate fundamentally different managerial responses and quality improvement strategies within the healthcare system [12,13].

## 2. The Purpose and the Objectives of the Study

This study addresses a central question in healthcare quality assessment: What concerns patients more—the core clinical aspects of treatment and diagnostics, or the organizational issues surrounding service delivery? To answer this, we introduce a novel machine learning approach that classifies patient complaints into two distinct, management-relevant categories.

The first category, medical content (M), encompasses dissatisfaction directly related to the clinical process. This includes complaints about the diagnosis of a disease, the prescribed treatment, and the result of a medical intervention on the patient’s health.

The second category, organizational support (O), includes all aspects related to the service delivery environment. Typical O complaints involve wasted time waiting in line or for test results, difficulties in scheduling an appointment, lack of convenient geographical access, high costs, staff rudeness, and lack of cleanliness in the clinic.

The primary objective is to identify the prevailing type of complaint (M or O) within a large dataset of negative patient reviews. This is achieved through the following specific objectives:

1. To develop and validate a machine learning model for the accurate classification of negative patient reviews into M and O categories.
2. To track the dynamics of M and O complaints over time to identify significant trends and structural shifts.
3. To compare the distribution of complaint types across different professional groups of doctors.
4. To assess the variation in complaint types among key demographic groups of patients, specifically by gender and across different major cities.

## 3. A Review of Research on Social Media-Based Healthcare Service Evaluations and Physician Reviews

The shift from traditional surveys to the automated analysis of patient-generated text from social media and review platforms represents a significant advancement in healthcare quality assessment [2,3]. This approach offers a more objective and representative picture of patient attitudes by leveraging large, independent samples. A key challenge in this domain is the development of intelligent text classification algorithms capable of handling the unique linguistic features of online reviews to categorize feedback based on criteria such as sentiment, target, and content [10,14].

The foundation of this research area is the concept of electronic word-of-mouth (eWOM), which refers to informal, internet-based communications about products and services [15,16,17]. eWOM, particularly in the form of online reviews, has become a highly influential source of information for consumers, a trend that extends fully to the healthcare sector [8,18,19]. The proliferation of Physician Rating Websites (PRWs) is a testament to this, with high adoption rates among both patients and doctors [1]. These platforms allow for the evaluation of individual doctors [2,10,14,20,21] and entire hospitals [12,13], though the focus of reviews can vary by country and context [22,23].

A substantial body of research utilizes machine learning to analyze the content and sentiment of these reviews. For instance, studies have identified prevalent topics and their associated emotions in reviews from the US [24] and Spain [25]. Beyond topic modeling, a critical finding is the potential real-world impact of these ratings; for example, one study linked higher surgeon ratings on a PRW to significantly better patient survival rates, suggesting that these platforms can guide patients toward higher-quality care [26].

However, the interpretation of patient reviews is complex and influenced by socio-demographic factors. Research indicates that a doctor’s gender, age, and specialty can affect the ratings they receive [4,5,27,28,29,30]. Similarly, patient characteristics, such as their age, insurance status, and geographic location, also play a role in shaping their feedback [4,5,31]. Furthermore, demographic factors influence not only the content of a review but also the likelihood of a patient writing one in the first place [6,9,32,33].

This study builds upon our previous work, where we developed a machine learning algorithm to classify patient reviews in Russian-language contexts [34]. While that model effectively categorized feedback for doctors versus clinics, the distinction between strictly medical (M) and organizational (O) shortcomings was not always precise. Therefore, the present study introduces a novel, management-oriented classification framework that directly distinguishes between complaints about “treatment/diagnostics” (M) and “service organization” (O). This refined approach is designed to provide healthcare managers with more actionable insights for targeted quality improvement, addressing a clear gap in the current literature on patient review analysis.

## 4. Database for Research

We use data from the review aggregator infodoctor.ru. The comparative advantage of this aggregator over alternative large Russian platforms (prodoctorov.ru, docdoc.ru) is the availability of grouping of reviews by rating from 1 to 5 stars in the context of different Russian cities, which significantly simplifies the data collection procedure.

A subsample of negative reviews was compiled (18,680 reviews, 1 star out of 5, the worst reviews). Of the subsample of negative reviews, 7425 reviews are related to Moscow, 2211 to St. Petersburg, and the remaining negative reviews are related to 14 other Russian cities with a population of over a million. The data were collected for the period from July 2012 to August 2023. For additional testing, a subsample from another Russian review aggregator website prodoctorov.ru (5037 reviews tagged by sentiment into negative and positive reviews) was used, as well as a subsample from the American review aggregator RateMDs.com (12,304 observations of the most negative sentiment, reviews translated into Russian). The American data was incorporated to augment the training set and to test the robustness of our classification model on a different cultural and healthcare context.

## 5. Research Methods

### 5.1. Data Annotation and Classification Model

Annotation Procedure. To create a labeled training set, we employed an independent annotation procedure. Three expert annotators independently classified each review into one of three categories: Medical content (M), pertaining to diagnosis, treatment, and outcomes; Organizational support (O), pertaining to logistics, cost, and communication; or Mixed (combined) content (C), containing elements of both. The final class for each review was assigned based on a majority vote (i.e., agreement by at least two annotators). This process was conducted on a preliminary Moscow sample of 8116 reviews from infodoctor.ru. After this annotation and the subsequent exclusion of reviews where a definitive M or O class could not be reliably assigned (e.g., those remaining in the C category or with no annotator agreement), the final, clean Moscow sample used for analysis contained 7425 reviews. This high-quality ground-truth dataset was used for model training.

Model Training and Selection. We trained and evaluated several machine learning models for the M/O/C classification task, including multiple Naive Bayes variants (ComplementNB, MultinomialNB, and BernoulliNB), Logistic Regression (LR), and Support Vector Machines (SVM). The training and testing sets were divided in a 90:10 ratio. The models were trained on a combined dataset to enhance robustness and generalizability. This dataset integrated the annotated reviews from the final Moscow sample with translated reviews from the United States (10,959 observations from RateMDs.com).

Rationale for Data Combination. Combining Russian and translated English reviews served a dual purpose: it increased the size of the training corpus and introduced greater linguistic diversity. This approach acts as a form of data augmentation, testing and reinforcing the model’s ability to learn the fundamental, transferable features of M and O complaints that are consistent across different healthcare systems and cultural contexts. The model’s primary application remained the classification of reviews from Russian cities, and its subsequent high performance on this target data confirms the effectiveness of this strategy in building a more robust classifier.

Final Model. After a comprehensive comparison, a model based on Logistic Regression demonstrated superior performance and was selected for the final classification. This model achieved an accuracy of 88.5% on a held-out test set. The confusion matrix for the logistic regression model is provided in the Results section (Table 1). It was subsequently used to classify the negative reviews from St. Petersburg and other Russian cities that were not part of the initial manual annotation.

### 5.2. Variable Construction

Patient Gender. Gender determination used a two-step protocol for reliability:

1. Manual Annotation: For the majority of reviews, gender was identified manually using clear markers such as the author’s name and gendered grammatical endings (e.g., verb and adjective forms in Russian).
2. LLM Imputation: For residual cases where manual classification was uncertain, we used the YandexGPT large language model (LLM) to predict gender. The quality of this automated classification was verified by comparing the thematic structure (distribution of M/O/C classes) of reviews in the manually labeled and LLM-imputed subsets; the consistent patterns observed between these subsets validated the imputation approach. Physician Specialty. The doctor’s specialization was available in the raw data. For a more meaningful and statistically robust analysis, these specializations were consolidated into six major groups: Gynecology, Dermatology, Neurology, Dentistry, Urology, and Functional Diagnostics.

## 6. Main Results of the Study

### 6.1. Model Performance and Classification Focus

We trained and evaluated multiple machine learning models on the combined dataset for the M/O/C classification task. The evaluated models included three variants of the Naive Bayes classifier (ComplementNB, MultinomialNB, BernoulliNB), Logistic Regression (LR), and Support Vector Machines (SVM). A hybrid algorithm based on a modal forecast from these models was also tested. The data was split into training and testing sets with a 90:10 ratio.

Logistic Regression achieved the highest accuracy among all tested models and was selected as the final classifier. The confusion matrix for the logistic regression model on the test set is presented in Table 1.

For the core analysis in this study, we focus on the distinction between purely medical (M) and organizational (O) complaints to derive clear, actionable insights for healthcare management. Consequently, reviews from the mixed (C) category were excluded from the subsequent comparative analysis across cities, genders, and specialties. This decision affected 4346 reviews, constituting approximately 23.3% of the annotated sample. We acknowledge that this choice simplifies a more complex reality and posit that future research could employ multi-label classification techniques to analyze the co-occurrence of M and O themes within a single review.

The model’s performance in distinguishing the primary classes of interest, M and O, was robust. The rate of confusion between these two classes was 11.5% (134 out of 1162 observations when considering only M and O predictions), indicating sufficient reliability of the classifier for the present analysis.

### 6.2. Temporal Dynamics of Complaint Types

**Figure 1:**
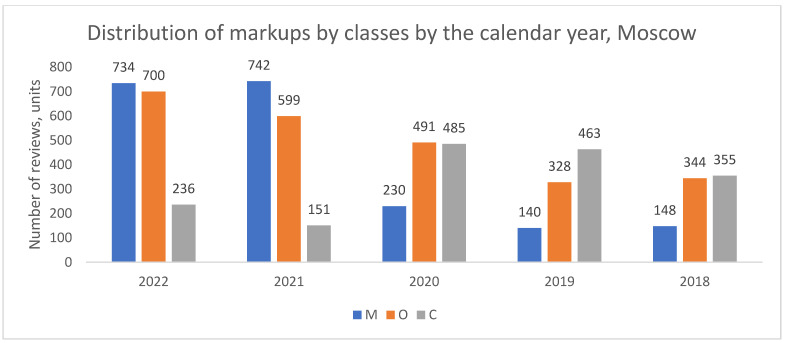
Distribution of review classes by calendar year, Moscow, 2018–2022. Notes: M—medical content, O—organizational support, C—mixed (combined) reviews (here and below).

The analysis of the classified reviews revealed a notable structural shift in the nature of patient complaints in Moscow over time (Figure 1). While organizational complaints (O) predominated in earlier years, a crossover occurred around 2021, after which medical complaints (M) became the prevalent category. This shift is visually evident in Figure 1 and represents a significant change in the pattern of patient dissatisfaction in the capital.

**Figure 2:**
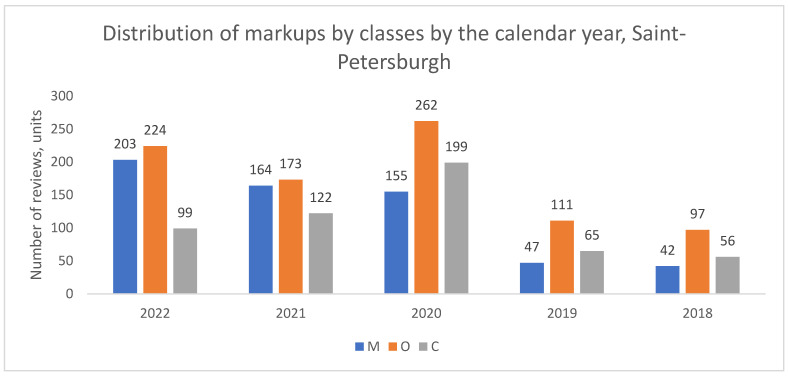
Distribution of review classes by calendar year, St. Petersburg, 2018–2022.

**Figure 3:**
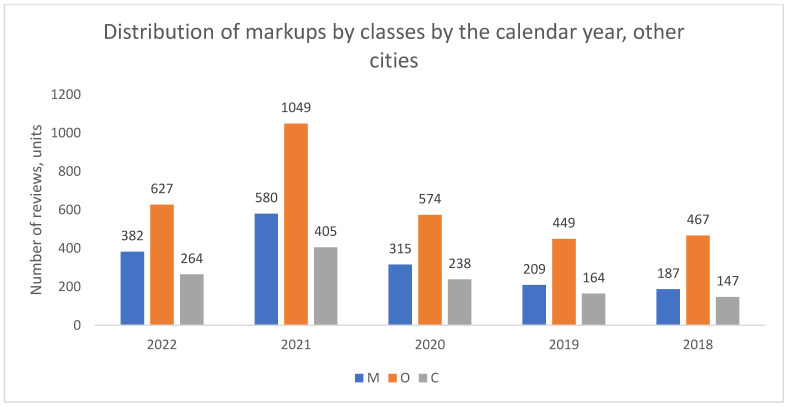
Distribution of review classes by calendar year, remaining 14 Russian cities with over a million inhabitants (combined), 2018–2022.

In contrast to Moscow, no such structural shift was observed in St. Petersburg (Figure 2) or in the aggregate of other million-plus cities (Figure 3). In these locations, organizational complaints (O) remained the dominant category throughout the entire study period from 2018 to 2022.

The identified shift in Moscow’s complaint structure after 2021 is a salient finding that warrants further investigation. We refrain from making causal inferences, as our data does not allow for testing specific hypotheses. However, this finding opens avenues for future research to investigate potential underlying factors, which could include changes in treatment protocols, shifts in the physician workforce, or evolving patient expectations, potentially influenced by the COVID-19 pandemic.

### 6.3. Analysis by Patient Gender

The distribution of complaint types was also analyzed by patient gender. The demographic composition of the review authors across cities is summarized in Table 2, showing a consistent overrepresentation of women, which is a common feature of such online platforms.

**Figure 4:**
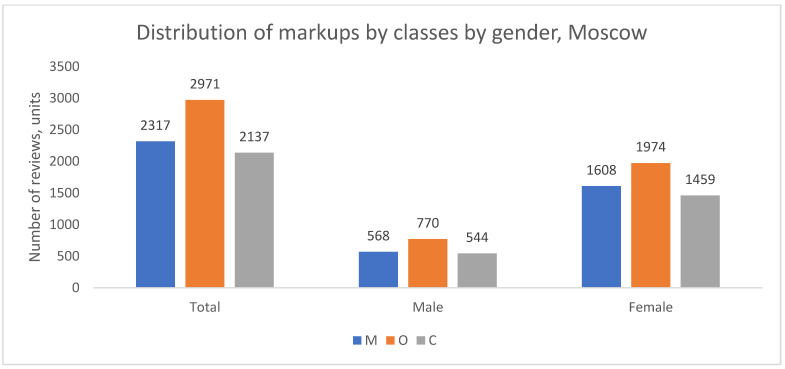
Distribution of review classes by patient gender, Moscow, 2012–2023.

**Figure 5:**
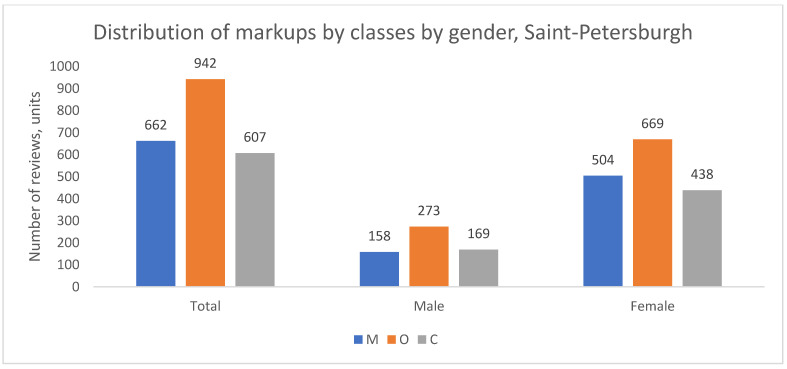
Distribution of review classes by patient gender, St. Petersburgh, 2012–2023.

**Figure 6:**
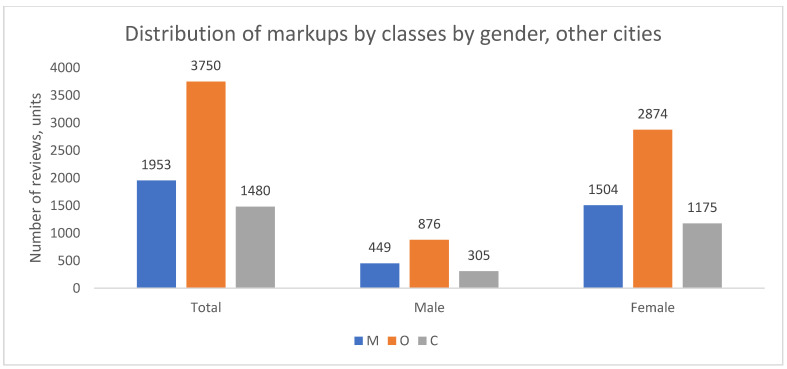
Distribution of review classes by patient gender, other cities, 2012–2023.

Despite this gender imbalance in review authorship, the thematic structure of complaints (the proportion of M to O) was remarkably similar between men and women within each geographical group, as shown in Figure 4, Figure 5 and Figure 6. In all cities, for both genders, organizational issues were the predominant reason for negative reviews. The key finding here is the disparity in the volume of feedback rather than a difference in the nature of the concerns raised.

In summary, while women author the majority of negative reviews across all cities, the fundamental structure of their complaints—predominated by organizational issues—does not differ substantially from that of men. This suggests that the core drivers of patient dissatisfaction are related to systemic or service-wide factors rather than gender-specific concerns.

## 7. Classification of Reviews by Physician Specialty

We analyzed the distribution of complaint types across the six largest medical specializations: gynecology, dermatology, neurology, dentistry, urology, and functional diagnostics.

**Figure 7:**
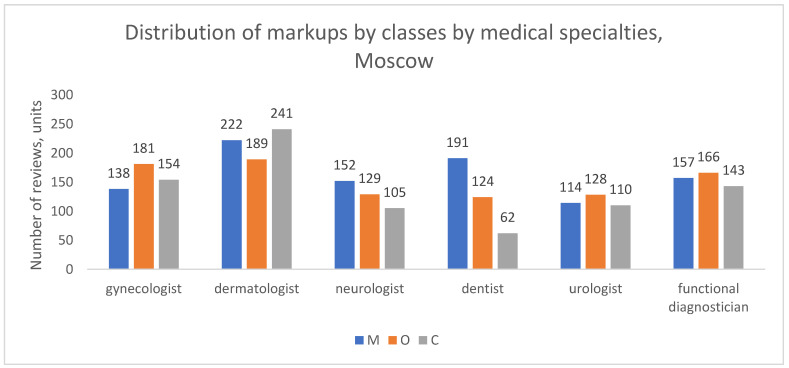
Distribution of review classes by medical specialties, Moscow, 2012–2023.

In Moscow (Figure 7), the pattern of complaints varied notably by specialty. Patients of dermatologists, neurologists, and dentists more frequently cited reasons related to medical content (M); for instance, among dentists, M reviews accounted for half of all classifiable complaints (50.7%, or 191 out of 377 reviews). Conversely, patients of gynecologists, urologists, and functional diagnosticians more often complained about organizational support (O); for example, among gynecologists, O reviews constituted 38.3% of all classifiable complaints (181 out of 473 reviews).

**Figure 8:**
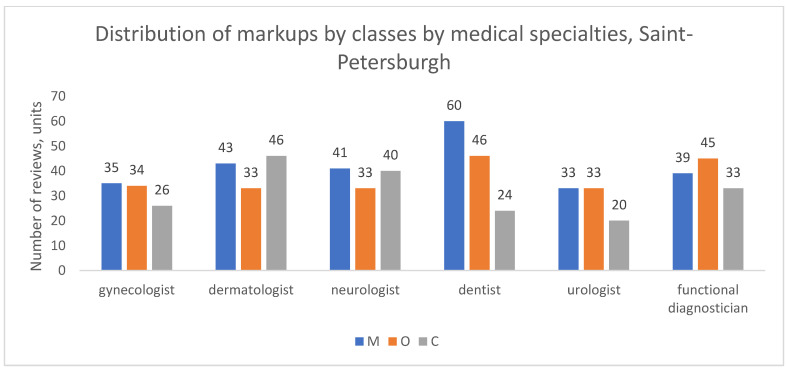
Distribution of review classes by medical specialties, St. Petersburgh, 2012–2023.

A distinct profile emerged in St. Petersburg (Figure 8). For most of the top-six specialties (with functional diagnostics and urology being the exception), complaints about medical content (M) were the most frequent category. For instance, among dentists, neurologists, and dermatologists, M reviews accounted for approximately 46%, 36%, and 35% of classifiable complaints, respectively. Despite this, organizational complaints (O) still predominated in the city’s overall dataset because the bulk of O reviews were concentrated in other medical specializations not included in this top-six analysis.

**Figure 9:**
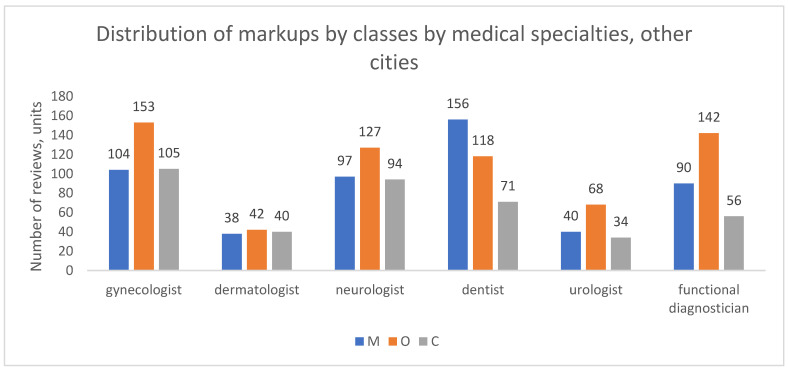
Distribution of review classes by medical specialties, other cities, 2012–2023.

In the aggregate of other cities (Figure 9), organizational complaints (O) were the most frequent reason for negative reviews across all specialties, with the exception of dentistry, where medical (M) and organizational (O) complaints were more balanced (45% and 34% of classifiable reviews, respectively). For other specialties, such as functional diagnostics, O reviews clearly dominated, constituting nearly half of all complaints (49%).

These findings demonstrate that the predominant type of patient dissatisfaction is not uniform across medical specialties and exhibits distinct geographic patterns. The specific clinical context and the local healthcare environment appear to shape whether patients’ primary concerns are related to medical outcomes or service organization.

## 8. Classification of Reviews by Patient Gender and Physician Specialty

To gain a more nuanced understanding, we constructed patient feedback profiles that combine gender and medical specialty. For this analysis, we used five specialty groups (excluding gynecology, as gynecologists typically serve only female patients). To clearly visualize the balance between complaint types, we calculated the ratio of M reviews to O reviews for each subgroup. A ratio greater than 1 indicates a predominance of medical complaints, while a ratio less than 1 indicates a predominance of organizational complaints.

**Figure 10:**
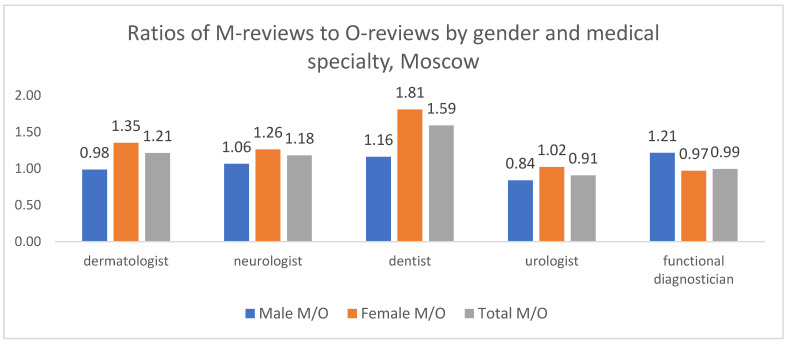
Distribution of M/O review ratios by patient gender and medical specialty, Moscow, 2012–2023. The absolute numbers of M and O reviews underlying these ratios are provided in Supplementary Table S1.

In Moscow (Figure 10), the M/O ratio reveals gender-specific patterns within certain specialties. Notable differences were observed in dermatology and urology, where the M/O ratio was higher for women than for men, indicating a greater relative focus on medical complaints among female patients in these fields. An inverse pattern was found in functional diagnostics, where men showed a higher M/O ratio. In neurology and dentistry, the M/O ratio was greater than 1 for both genders, indicating a general predominance of medical complaints.

**Figure 11:**
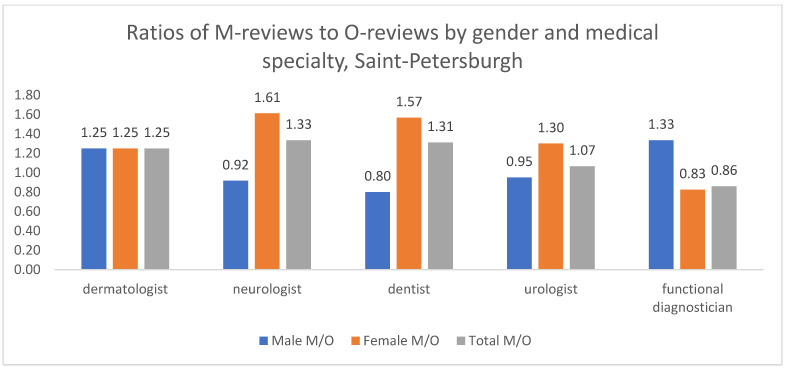
Distribution of M/O review ratios by patient gender and medical specialty, St. Petersburg, 2012–2023. The absolute numbers of M and O reviews underlying these ratios are provided in Supplementary Table S1.

In St. Petersburg (Figure 11), the authors of M reviews were predominantly women across most specialties, as indicated by their higher M/O ratios. The exception was functional diagnostics, where men had a higher M/O ratio. It is important to note that in absolute terms, O reviews for functional diagnostics were more numerous, as they were authored primarily by women, who constitute the majority of the sample.

**Figure 12:**
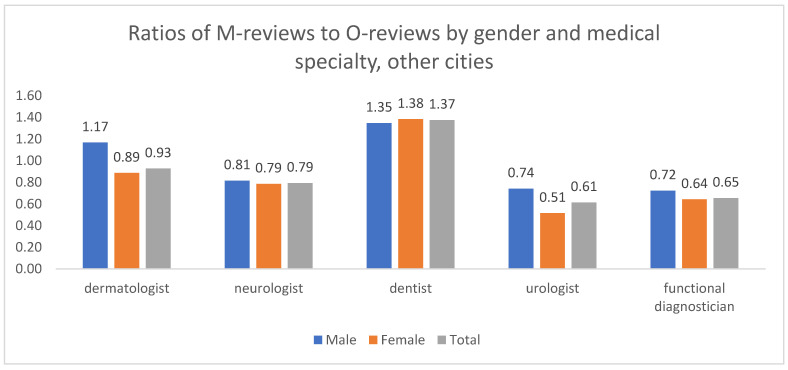
Distribution of M/O review ratios by patient gender and medical specialty, other cities, 2012–2023. The absolute numbers of M and O reviews underlying these ratios are provided in Supplementary Table S1.

For the aggregate of other cities (Figure 12), the M/O ratios were largely similar for men and women across most specialties, suggesting no strong gender-based differences in complaint focus. A notable exception was dermatology, where the M/O ratio was greater than 1 for men but less than 1 for women, indicating that male patients in this specialty tended to focus on medical issues, while female patients focused more on organizational ones.

In summary, the interplay between patient gender and physician specialty reveals complex, city-specific patterns in the focus of patient complaints. While gender differences in complaint structure were minimal in the aggregate of smaller cities, they were pronounced in the two capitals. In Moscow, the direction of these differences varied by specialty, whereas in St. Petersburg, women consistently showed a greater tendency toward medical complaints across most of the analyzed specialties. These findings underscore that a multidimensional analysis, considering both demographic and professional factors, is crucial for accurately interpreting patient feedback and tailoring targeted quality improvement interventions.

## 9. Classifying Patient Reviews by Gender Using Generative Artificial Intelligence

This section details the development of a method for determining patient gender from review text, a necessary step for platforms that do not collect this demographic data. We utilized generative artificial intelligence (YandexGPT) for this task. A sample of 4000 negative reviews (1 star) from Moscow clinics (2012–2023) was used for this analysis.

### 9.1. Prompt Engineering and Model Selection

We designed and tested three distinct prompts for YandexGPT, with varying levels of detail and instruction, to optimize gender prediction. The prompts are detailed in Table 3. The high baseline accuracy across all prompts (~85%) suggests that the underlying YandexGPT model possesses a strong inherent capability for gender classification from Russian text, likely due to its pre-training on large corpora where grammatical gender is a prominent feature. Consequently, the additional instructions (focusing on verb endings and providing examples) provided only marginal, non-systematic improvements, as the model had largely already learned these cues.

Given the minimal performance differences, we selected a hybrid approach (Y_MODE) for final application. This method assigns gender based on the modal prediction from the three individual prompts (Y_1, Y_2, Y_3). This ensemble strategy was chosen for its potential robustness and stability, as it mitigates the risk of relying on a single, potentially suboptimal prompt and leverages the consensus across multiple approaches.

### 9.2. Validation and Application

**Figure 13:**
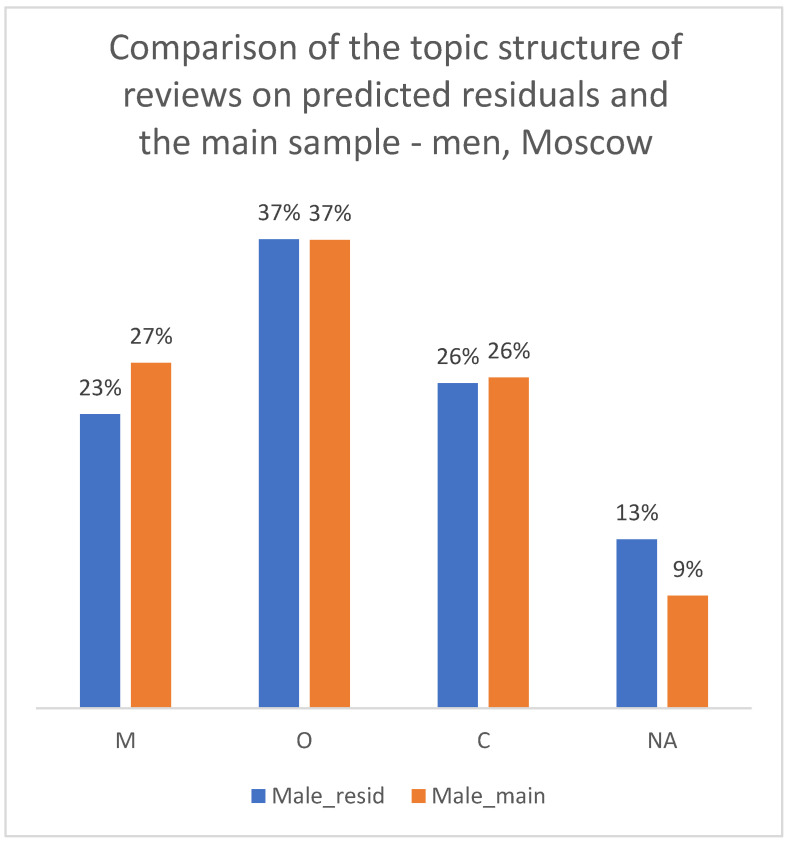
Comparison of the topic structure of reviews in the main sample and the sample from residual observations, 2012–2023. Men, Moscow.

**Figure 14:**
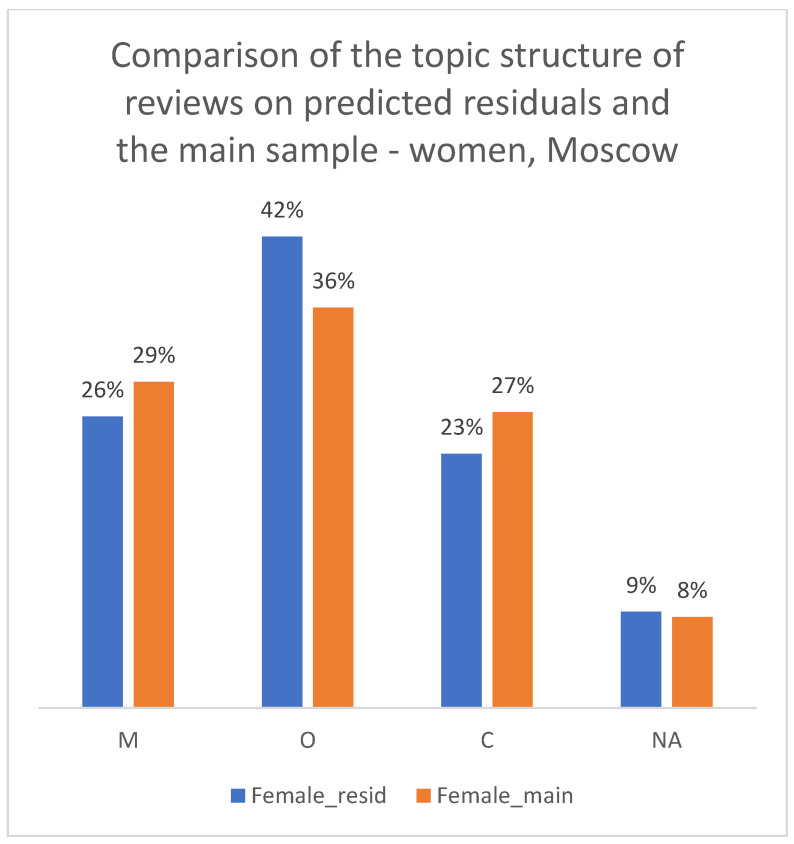
Comparison of the topic structure of reviews in the main sample and the sample from residual observations, 2012–2023. Women, Moscow.

**Figure 15:**
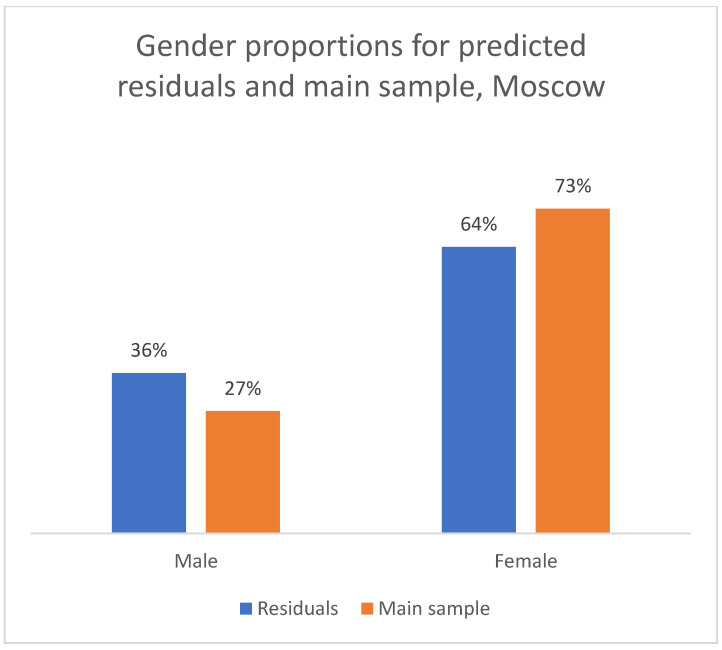
Comparison of the topic structure of reviews in the main sample and the sample from residual observations, 2012–2023. Gender proportions for predicted residuals and main sample, Moscow.

We applied the hybrid (Y_MODE) algorithm to the residual sample of reviews with previously unknown gender. To validate the quality of this imputation, we compared the thematic structure (distribution of M/O/C classes) between the original manually labeled sample and the new LLM-imputed sample. As shown in Figure 13, Figure 14 and Figure 15, the proportions of complaint types were very similar between the two groups for both men and women. This consistency in the resulting thematic profiles, rather than a direct measure of gender accuracy, serves as a successful proxy validation of the imputation method for the purposes of our subsequent analysis.

In conclusion, we successfully developed and validated a robust method for inferring patient gender from Russian-language medical reviews using generative AI. The high baseline performance of YandexGPT for this task indicates its strong capability for this specific natural language understanding problem in Russian. The hybrid ensemble approach (Y_MODE) was established as the most reliable strategy. Most importantly, the validation confirmed that the gender imputation process did not distort the underlying thematic structure of the feedback. This methodology provides a valuable tool for enriching demographic analysis of patient reviews from platforms where such data is not explicitly provided, thereby expanding the potential for socio-demographic research in digital healthcare.

## 10. Comparison of Medical Review Structures in Russia and the USA (Additional Task)

We conducted a parallel analysis on a dataset of American reviews from RateMDs.com. This served a dual purpose: to test the robustness of our classification algorithm on data from a different cultural and healthcare context, and to enable a comparative analysis of complaint structures between the two countries.

The US dataset comprised 12,304 highly negative reviews (rated 2 stars or lower) across five major specialties (dentistry, dermatology, gynecology, neurology, urology) from 2018 to 2024. Patient gender was not available in the initial data and was imputed using a simplified YandexGPT prompt tailored to the English language. The annotation procedure for classifying reviews into M, O, C, and NA categories mirrored the protocol used for the Russian data.

**Figure 16:**
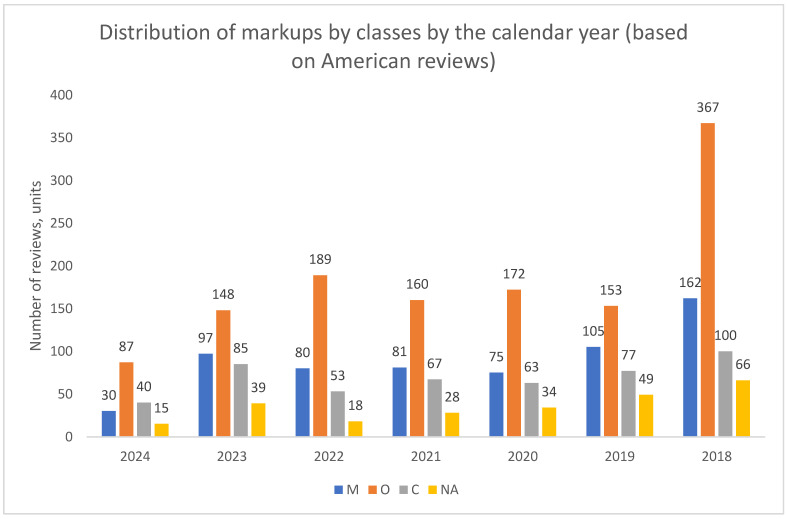
Distribution of class markups by calendar year, United States, 2018–2024. Note: NA—the content of the review is not clearly defined.

**Figure 17:**
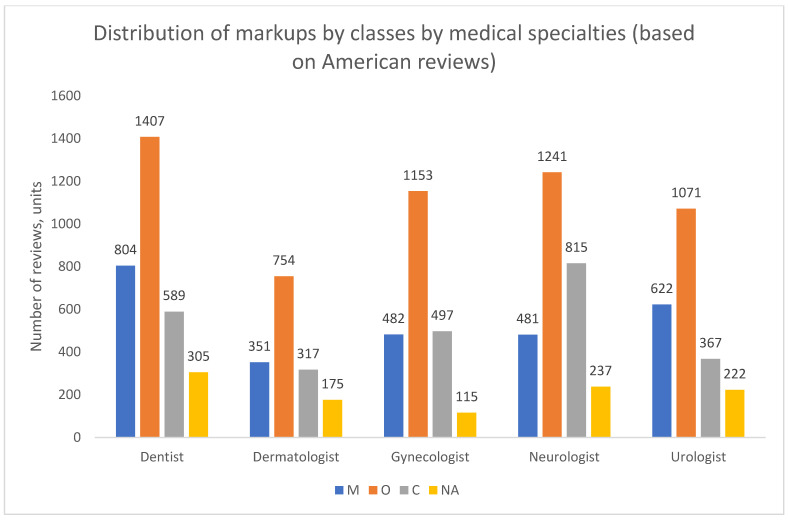
Distribution of class markups by medical specialization, United States, 2018–2024. Note: NA—the content of the review is not clearly defined.

**Figure 18:**
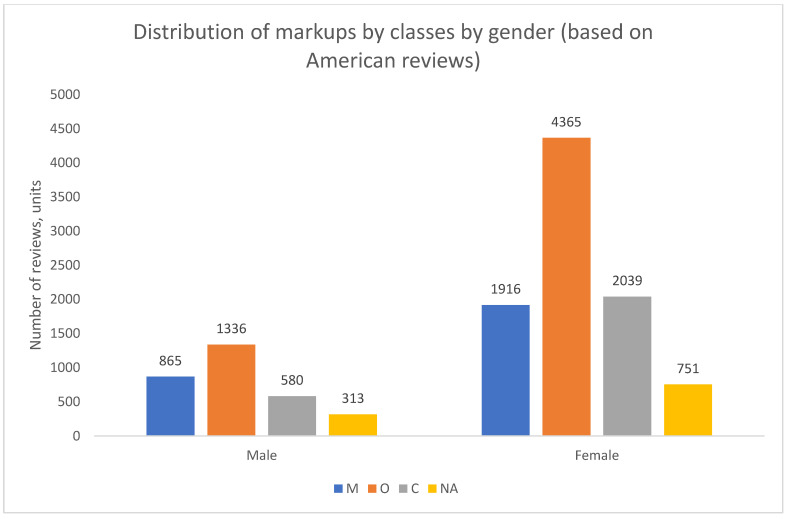
Distribution of class markups by patient gender, United States, 2018–2024. Note: NA—the content of the review is not clearly defined.

The results from the American data reveal a strikingly consistent pattern (Figure 16, Figure 17 and Figure 18). In contrast to the dynamic and varied structures observed in Russian cities, the US data shows no significant temporal shifts or major variations across medical specialties. Organizational complaints (O) overwhelmingly predominate in all analyzed dimensions—over time, across specialties, and for both genders.

**Figure 19:**
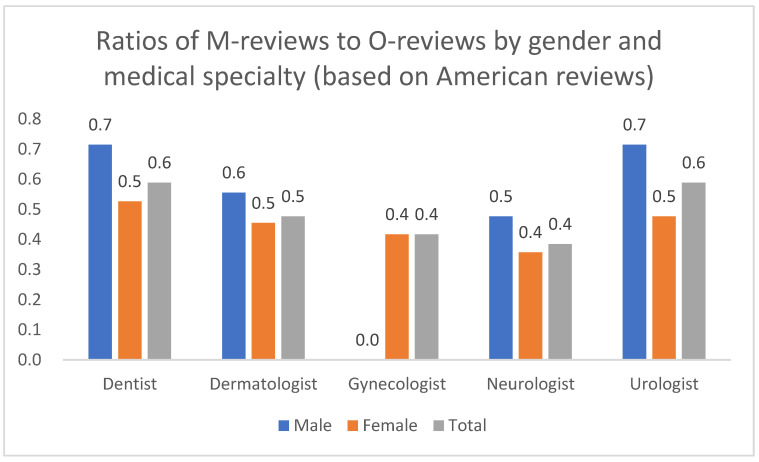
Distribution of M/O review ratios by patient gender and physician specialty, United States, 2018–2024.

To ensure a direct and consistent comparison with the Russian results presented in Section 8, we calculated the ratio of M-reviews to O-reviews for the US data (Figure 19). A ratio below 1.0 indicates a predominance of organizational complaints. The findings are unequivocal: the M/O ratio remains below 1.0 for all subgroups, confirming the absolute dominance of O-type complaints in the US sample. This stands in sharp contrast to the more mixed and geographically dependent patterns found in Russia, particularly in Moscow and St. Petersburg. Furthermore, the M/O ratio is consistently lower for American women than for men across all specialties, indicating that the predominance of organizational concerns is even more pronounced among female patients in the US.

In summary, the comparative analysis yields two key findings. First, it successfully demonstrates the robustness and cross-cultural applicability of our classification algorithm. Second, it reveals a fundamental difference in the nature of patient dissatisfaction: while organizational issues are a major concern everywhere, they define the negative feedback landscape in the United States to a much greater extent than in Russia, where medical-content complaints play a significant and sometimes dominant role, especially in the two largest cities.

## 11. Key Findings of the Study

1. We developed and validated a robust machine learning framework for classifying negative patient reviews into management-relevant categories: medical content (M) and organizational support (O). The final logistic regression model achieved an accuracy of 88.5%, providing a reliable tool for automated analysis.
2. A significant structural shift in patient complaints was identified in Moscow, where medical complaints (M) began to prevail over organizational ones (O) starting in 2021. This trend was unique to the capital and not observed in St. Petersburg or other major Russian cities, where organizational complaints remained dominant throughout the study period.
3. The distribution of complaint types varied substantially by medical specialty and city. In Moscow, patients of dermatologists, neurologists, and dentists focused more on medical content, while patients of gynecologists, urologists, and functional diagnosticians more frequently cited organizational issues. In St. Petersburg, M reviews were more common within the leading specialties, despite O reviews prevailing overall. In other cities, organizational complaints were consistently dominant across almost all specialties.
4. Gender differences in complaint focus were most pronounced in St. Petersburg, where women were more frequently the authors of M reviews and men of O reviews across leading specialties. In contrast, no significant gender-based differences in complaint structure were found in Moscow or the aggregate of other cities.
5. The methodology was successfully tested on international data, confirming its robustness. A comparative analysis with US reviews revealed a fundamental cross-country difference: organizational complaints (O) demonstrated an absolute and consistent predominance in the United States across all years, specialties, and gender groups, with this pattern being especially pronounced among women. This contrasts with the more heterogeneous and dynamic structures found in Russia.

## 12. Discussion and Prospects

This study developed and applied a novel machine learning framework to classify patient complaints from online reviews into medical (M) and organizational (O) categories, revealing significant geographic, temporal, and demographic variations in Russia and cross-cultural differences with the United States. Our discussion integrates these findings with the existing literature, acknowledges the study’s limitations, and outlines avenues for future research.

### 12.1. Interpretation of Key Findings

Our core finding—the distinct complaint structures across Russian cities—highlights the importance of regional context in healthcare quality assessment. The shift in Moscow since 2021, where medical complaints began to prevail, is particularly noteworthy. While the data does not permit causal inference, this trend could reflect several factors, such as post-pandemic changes in patient expectations regarding treatment outcomes [35], evolving medical protocols, or shifts in the capital’s healthcare system. The stark contrast with St. Petersburg and other cities underscores that drivers of patient dissatisfaction are not uniform and require localized management strategies.

The robust performance of our classification model (88.5% accuracy) demonstrates the viability of automated tools for processing patient feedback at scale. This aligns with a growing body of research that uses NLP for healthcare quality monitoring [2,10,14], but advances it by introducing a management-oriented M/O dichotomy. The successful application of this model to US data further confirms its robustness and cross-cultural applicability, revealing a fundamental difference: an overwhelming predominance of organizational complaints in the US compared to the more varied Russian landscape. This cross-national divergence may reflect systemic differences in healthcare delivery, financing, and patient expectations between the two countries [23].

### 12.2. Limitations

Several limitations of this study should be acknowledged. First, our analysis relied on negative reviews from specific online aggregators, which may not be fully representative of the entire patient population, as users of such platforms can possess distinct demographic characteristics [6,32]. Second, the exclusion of the mixed (C) category from the comparative analysis, while methodologically justified for clarity, resulted in the loss of nuanced data; future work could benefit from multi-label classification approaches. Third, the gender classification, while validated thematically, was imputed for a portion of the dataset using an LLM, which, despite its high accuracy, is an indirect method. Finally, and most notably, our ability to classify reviews by patient age was limited, with accuracy not exceeding 62%. This is a common challenge in the field due to the lack of explicit linguistic age markers in short texts and the lower digital footprint of older adults [36,37], representing a significant gap for future methodological work.

### 12.3. Future Research Directions

Building on this work, future research should prioritize the development of more sophisticated methods for demographic inference, particularly for age. Exploring multi-modal data (e.g., combining text with platform metadata) or transfer learning from platforms with known user demographics could be promising paths. The hypotheses generated by our findings, especially the shift in Moscow, warrant dedicated investigation using mixed-methods approaches, potentially linking review data with healthcare system statistics or conducting qualitative patient interviews. Finally, applying the M/O classification framework to longitudinal data and in other national contexts could provide deeper insights into the evolution and global drivers of patient satisfaction.

## 13. Conclusions

This study developed and validated a robust machine learning framework for the automated classification of patient reviews into management-actionable categories: medical content (M) and organizational support (O). The logistic regression model achieved high accuracy (88.5%), providing a reliable tool for large-scale analysis. Our analysis of a substantial dataset of Russian-language reviews revealed that the structure of patient dissatisfaction is not monolithic but exhibits significant geographic variation. The most striking finding was the distinct structural shift in Moscow, where medical (M) complaints began to prevail over organizational (O) ones after 2021, a trend not observed in other major Russian cities.

Furthermore, the study uncovered specific patterns linked to medical specialties and patient gender, particularly in St. Petersburg, demonstrating the value of a multi-dimensional analytical approach. The successful application of the algorithm to US review data confirmed its cross-cultural robustness and highlighted a fundamental difference: an overwhelming predominance of organizational complaints in the American healthcare context compared to the more heterogeneous picture in Russia.

The developed algorithm provides a practical and scalable tool for healthcare management, enabling regional and specialty-specific monitoring of patient feedback. By distinguishing between clinical and service-related issues, it allows healthcare administrators to target quality improvement efforts more precisely. The implementation of such automated monitoring systems can facilitate a more responsive, patient-centered healthcare system by providing real-time, actionable insights directly derived from patient experiences.

## Figures and Tables

**Table 1:** Confusion Matrix for the Logistic Regression Model (Test Set).

	Predicted M	Predicted O	Predicted C
Actual M	339	94	117
Actual O	40	689	102
Actual C	148	159	220

Source: compiled by the authors (here and below).

**Table 2:** Distribution of reviews by gender in Russian cities, 2012–2023.

	Moscow	Saint Petersburgh	Other Cities
Share of women	74.7%	72.9%	77.3%
Share of men	25.3%	27.1%	22.7%

**Table 3:** Description of prompts for determining patient gender and the values of quality metrics.

Variable	Description	Comment	Result Accuracy	F1-Score Result
**TEXT**	Original text of the comment			
**GENDER**	Actual data on the gender of patients (1—male, 2—female)			
**Y_1**	Predicted data on the gender of patients based on the prompt: “Determine the gender of the authors of the comments. Present the results as a table of 2 columns: the first is the number of the comment, the second is the gender of the author (male or female).”	Basic request without additional input	**0.8552**	**0.7343**
**Y_2**	Predicted data on the gender of patients based on the prompt: “Determine the gender of the authors of the comments. Present the results in the form of a table of 2 columns: the first is the number of the comment, the second is the gender of the author (male or female). First of all, pay attention to the endings of the verbs indicating belonging to the male or female gender.”	Adding an instruction to look at the word endings	**0.8572**	**0.7427**
**Y_3**	Predicted data on the gender of patients based on the prompt: “Determine the gender of the authors of the comments. Present the results in the form of a table with 2 columns: the first is the number of the comment, the second is the gender of the author (male or female). First of all, pay attention to the endings of the verbs indicating belonging to the male or female gender. Example: “I did this” (in Russian: «*Я сделал**а** это*»)—the author of this comment is a woman, this is evident from the form of the verb. “I did this” (in Russian: «*Я сделал это*»)—the author of this comment is a man.”	Adding an example of gender endings	**0.8542**	**0.7141**
**Y_MODE**	Predictive data on patient gender based on a hybrid approach of three predictions	Modal value from Y_1, Y_2, Y_3	**0.8585**	**0.7380**

Note: Bold is used for variable names and to highlight the best performance metrics in each column. In the example for prompt Y_3, bold is used in the Russian phrases «Я сделала» and «Я сделал» to highlight the critical grammatical endings that indicate the author’s gender.

## Data Availability

The raw data supporting the conclusions of this article were derived from publicly available sources (infodoctor.ru, RateMDs.com) under their respective terms of service. The processed datasets and code used for analysis are available from the corresponding author upon reasonable request.

## Supplementary Materials

The following supporting information can be downloaded at: https://www.mdpi.com/article/10.3390/healthcare13202641/s1, Table S1. Absolute counts of M and O reviews underlying the M/O ratios presented in Figure 10, Figure 11, Figure 12 and Figure 19.

## Abbreviations

The following abbreviations are used in this manuscript:

eWOM	Electronic Word of Mouth
PRW	Physician Rating Websites
ML	Machine Learning
LR	Logistic Regression
NLP	Natural Language Processing
LLM	Large Language Model
AI	Artificial Intelligence
GAI	Generative Artificial Intelligence
M	Medical Content (complaints about treatment/diagnosis)
O	Organizational Support (complaints about service organization)
C	Mixed (Combined) Content (both medical and organizational complaints)
