# Supplementary material for: Medical vs. Organizational Complaints: A Machine Learning Analysis Reveals Divergent Patterns in Patient Reviews Across Russian Cities
Source: Healthcare (Basel). 2025 Oct 20;13(20):2641. doi: 10.3390/healthcare13202641 (PMC12563190; doi:10.3390/healthcare13202641)
Supplement: Supplementary file 1 [file healthcare-13-02641-s001.zip › healthcare-3892722-supplementary.pdf]

healthcare

Supplementary Table S1. Absolute counts of M and O reviews underlying the M/O ratios presented in Figures 10, 11, 12, and 19.

City / Group	Physician Specialty	Patient Gender	Count of M-Reviews	Count of O-Reviews	M/O Ratio
Moscow	Dermatologist	Male	64	65	0.98
Moscow	Dermatologist	Female	146	108	1.35
Moscow	Neurologist	Male	50	47	1.06
Moscow	Neurologist	Female	87	69	1.26
Moscow	Dentist	Male	43	37	1.16
Moscow	Dentist	Female	132	73	1.81
Moscow	Urologist	Male	62	74	0.84
Moscow	Urologist	Female	46	45	1.02
Moscow	Functional Diagnostician	Male	17	14	1.21
Moscow	Functional Diagnostician	Female	132	136	0.97
St. Petersburg	Dermatologist	Male	10	8	1.25
St. Petersburg	Dermatologist	Female	30	24	1.25
St. Petersburg	Neurologist	Male	11	12	0.92
St. Petersburg	Neurologist	Female	29	18	1.61
St. Petersburg	Dentist	Male	12	15	0.80
St. Petersburg	Dentist	Female	47	30	1.57
St. Petersburg	Urologist	Male	19	20	0.95
St. Petersburg	Urologist	Female	13	10	1.30
St. Petersburg	Functional Diagnostician	Male	4	3	1.33
St. Petersburg	Functional Diagnostician	Female	24	29	0.83
Other cities	Dermatologist	Male	7	5	1.4
Other cities	Dermatologist	Female	30	35	0.86
Other cities	Neurologist	Male	21	27	0.78
Other cities	Neurologist	Female	64	83	0.77
Other cities	Dentist	Male	33	26	1.27
Other cities	Dentist	Female	112	80	1.4
Other cities	Urologist	Male	20	27	0.74
Other cities	Urologist	Female	17	35	0.49
Other cities	Functional Diagnostician	Male	13	14	0.93
Other cities	Functional Diagnostician	Female	54	85	0.64
United States	Dentist	Male	280	382	0.73
United States	Dentist	Female	524	1019	0.51
United States	Dermatologist	Male	106	193	0.55
United States	Dermatologist	Female	253	560	0.45
United States	Gynecologist	Male	-	-	-

United States	Gynecologist	Female	482	1153	0.42
United States	Neurologist	Male	149	309	0.48
United States	Neurologist	Female	332	931	0.36
United States	Urologist	Male	321	436	0.74
United States	Urologist	Female	300	633	0.47

***Note**: This table provides the raw data for the M/O ratios visualized in the figures. The ratio is calculated as Count of M-Reviews / Count of O-Reviews. Data for the 'C' (Mixed) category is not included in this calculation. The specialty "Functional Diagnostician" was not included in the U.S. analysis due to poor data representation and conceptual misalignment with the Russian healthcare system. Gynecologists in the U.S. sample predominantly serve female patients, hence the absence of data for males.*
